# Impacts of commercial bile acids on growth performance, immune responses and expression genes of lipid metabolism in Nile tilapia fingerlings *Oreochromis niloticus*

**DOI:** 10.1038/s41598-025-06813-0

**Published:** 2025-06-20

**Authors:** Yasser Marzouk, Mohamed Abdullah Zaki, Abd-Elaziz M. Nour, Ahmed Ismail Mehrim, Hala Saber Khalil

**Affiliations:** 1 Animal Production Department, Faculty of Agriculture, Alexandria University, Alexandria, Egypt; 2 Animal Production Department, Faculty of Agriculture, Mansoura University, Mansoura, Egypt; 3 College of Fisheries and Aquaculture Technology, Arab Academy for Science, Technology and Maritime Transport (AASTMT), Alexandria, 1029 Egypt

**Keywords:** Antioxidants, Digestive enzyme, Intestinal and liver morphology, Intestinal microbiota, Immunology, Physiology

## Abstract

The current investigation evaluated the impact of the dietary addition of commercial bile acids (BAs) on growth, nutrient assimilation, immunity, antioxidant status, intestinal and hepatic histomorphometry, and gene expression of lipid metabolism in Nile tilapia (*Oreochromis niloticus*). In a study conducted for seventy days, 180 healthy fingerlings weighing 9 ± 0.5 g were divided into 18 hapas measuring 0.7 × 0.7 × 1.0 m. The fish were fed on six meals enriched with varied amounts of BAs: 0.0 (D1), 0.1 (D2), 0.2 (D3), 0.3 (D4), 0.4 (D5), and 0.5 (D6) g/kg diet. Nile tilapia fed the D3 diet exhibited significantly enhanced growth performance, with a specific growth rate of 1.89%/day and had the greatest feed conversion ratio (1.25), protein productive value, and energy utilization (33.28%). Fish fed the D3 exhibited significantly the highest crude protein content (64.50%). Energy content varied significantly, with D1 showing the lowest value (533.34 Kcal/100 g) and D3 the highest (604.27 Kcal/100 g). D3 improved biochemical indicators, immunological parameters, and digestive enzymes of *O. niloticus*. Histological analysis revealed notable liver and intestinal integrity enhancements among fish receiving BA-enriched diets, especially D3. Additionally, gene expression related to lipid metabolism in liver, peritoneal fat, and muscle tissues was upregulated in the treatment groups, especially 0.2 g/kg BAs compared to the control group. Results illustrate significant modulation of lipid metabolism gene expression parameters (Adipose triglyceride lipase; ATGL, Hormone-sensitive lipase; HSL, Peroxisome proliferator-activated receptor α; PPARα, Fatty acid synthase; FAS) by BAs treatments and were upregulated in BA-fed groups (D2–D6). Conversely, Carnitine palmitoyl transferase 1; CPT^-1^and Insulin-like growth factor^-II^; Igf^-II^ expression declined, particularly when the BAs dose was increased. Accordingly, dietary 0.2 g/kg BAs supplementation positively influences on physiological, biochemical parameters, and lipid metabolic of Nile tilapia, making it a promising feed additive for aquaculture.

## Introduction

Aquaculture has become the primary means of increasing fish production in recent years, compensating for the decline in capture fisheries yields; aquaculture expansion has contributed to a 26.9% rise in per capita fish consumption^1^. In the last several centuries, the country’s aquaculture sector has grown substantially, with Nile tilapia (*Oreochromis niloticus*) emerging as the dominant species in production. This species alone accounts for approximately 9% of the world’s total aquaculture output^2^.

Several biological and economic traits make tilapia particularly well-suited for aquaculture. These fish exhibit rapid growth rates, adaptability to various environmental conditions, efficient feed conversion, and the ability to thrive on artificial diets^3^. Additionally, they are easy to breed in captivity, have high disease resistance, and have high fecundity. Their favorable meat quality and competitive market price further enhance their commercial value^4^. Another significant advantage of tilapia is its ability to be cultivated in diverse aquaculture systems, including semi-intensive and intensive methods and monoculture or polyculture setups^5,6^. It can also be farmed in freshwater and seawater environments across tropic, subtropic, and temperate regions^7^. Due to these attributes make Nile tilapia an essential commercial fish species globally, particularly in Egypt^8,9^.

Feed costs represent a significant portion of aquaculture production expenses, accounting for at least 60% of variable costs, with protein being the most costly component of aquafeeds^10^. Consequently, reducing the cost of dietary is a crucial strategy for mitigating the financial burden associated with rising feedstock prices. Two mathematical approaches have been proposed to achieve this objective. The first involves moderately lowering the inclusion levels of protein in aquafeeds while increasing the use of alternative energy sources to protein, including lipids and carbohydrates. These macronutrients have a “protein-sparing effect” and are more cost-effective, making them suitable alternatives for reducing protein dependency in aquaculture diets^11,12^. The second approach emphasizes replacing high-cost protein sources, such as fishmeal, with more affordable plant-based proteins^3^.

Despite these cost-saving measures, research has highlighted the potential drawbacks of both approaches. Prolonged consumption of diets rich in carbohydrates and lipids has been linked to abnormal hepatic lipid accumulation and metabolic disorders, which could adversely influence the growth efficiency and overall well-being of aquatic animals^13–15^. Similarly, excessive reliance on plant protein sources has been shown to cause several adverse impacts, such as diminished fish growth efficiency, decreased feed utilization, impaired lipid metabolism, disruptions in bile acid balance, and compromised liver function. The anti-nutritional properties of plant-based feed components are thought to be responsible for these negative effects^16,17^. Given these challenges, fish dietary professionals are increasingly exploring functional feed additives that are readily available and capable of enhancing growth performance, reducing metabolic and detoxification stress on the liver, and improving the overall health of farmed fish.

Bile acids, derived from cholesterol in the liver and deposited as bile salts, facilitate lipid digestion in fish through emulsification^18,19^. They activate lipases, promoting lipid breakdown, absorption, and transport^13,17^. Additionally, bile acids function as a signal, controlling the absorption and utilization of glucose and lipids to keep the body’s metabolic rate constant^20^.

The latest research has further emphasized the broader physiological benefits of bile acid supplementation in fish. Research indicates that nutritional inclusion of bile acids can enhance antioxidant capability^21^, exert anti-inflammatory effects^22^, strengthen immune responses^23^, and support intestinal status^24^. Given these multifaceted functions, the present study aims to assess the impact of commercial bile acid (BA) as a feed supplement with different levels on growth metrics, feed efficiency, proximate body compositions, innate immunity, antioxidant enzymes, histopathology of liver and intestine in addition to intestinal microbiota, and genes expression evaluation of lipid metabolism in hepatic, peritoneal fat, and muscle tissues in Nile tilapia, *O. niloticus* fingerlings.

## Materials and methods

### Ethical declaration

The current investigation was done following ethical considerations, adhering to the guidelines established by an ethics committee for animal care, and all trial approaches were permitted by the Institutional Animal Care and Use Committee (IACUC) at Alexandria University, Alexandria, Egypt. Approval number: (AU: 08/20/07/20/2/68). Every approach used in this study adhered to the applicable ethical standards and regulations, including the ARRIVE standards (https://arriveguidelines.org*).* The mono-sex Nile tilapia fingerlings were obtained with appropriate consent from the freshwater farm in Kafr El-Sheikh, Egypt, and experimental procedures at the Baltim Research Station were authorized accordingly.

### Investigational diet

Commercial bile acids were procured from Shang Dong Longchang Animal Health Product Co., Ltd, Jinan, China, primarily including 699.2 g BAs kg^–1^ hyodesoxycholic acid (HDCA), 189.2 g BAs kg^–1^ chenodeoxycholic acid, and 77.5 g BAs kg^–1^ hyocholic acid as determined by HPLC. Six experimental diets were prepared to evaluate the influence of bile acid addition, each containing isonitrogenous (30.44%) and isocaloric (437.42 Kcal/100 g diet). The diets included a control diet (D1) without bile acid dietary inclusion and five treatment diets (D2, D3, D4, D5, and D6), which were supplemented with 0.1, 0.2, 0.3, 0.4, and 0.5 g of commercial bile acids per kilogram of feed, respectively. These dietary formulations were designed to assess the probable benefits of bile acid supplementation as a feed additive for *O. niloticus* fingerlings.

The trial diets were formulated utilizing various ingredients bought from the local market, as shown in Table 1. All components were first ground, accurately weighed, and thoroughly melded for 10 min. Sunflower oil was then added, followed by another 10 min of mixing. To achieve the desired consistency, distilled warm water (350 ml kg^−1^) was gradually incorporated with the commercial bile acid and introduced to the feed components by consistent spraying and mixed effectively for thirty minutes until a firm dough was formed. This mixture was then processed using a mincer (Fama, TI 32 R, Italy), producing pellets with a diameter of 3.0 mm × 2 mm. The formulated diets were processed using a milling machine and subsequently air-dried for 48 h to ensure proper dehydration. Once dried, the diets were shaped into uniform pellets suitable for feeding the investigational fish.

**Table 1:** The configuration of the trial basal diet.

Items- Feed ingredients (g 100 g^−1^)	Trial diet
Soybean meal (CP 44)	22
Rice bran	12
Corn gluten meal (CP 60)	8
Yellow corn	35
Fish meal (CP 65)	18
Sunflower Oil	3
Vit. and Min.mix^1^	2
Proximate chemical analysis (%) on dry matter (DM) basis	
Dry matter	90.50
Crude Protein	30.44
Ether Extract	5.40
Ash	6.80
Crude Fiber	5.32
NFE	52.04
GE (Kcal/100 g)^2^	437.42
P/E ratio^3^ (mg protein kcal gross energy^−1^)	69.59

^1^ Vitamin-mineral mix (per kg): Vitamin; A (7,500,000 IU), B1 (650 mg), B2 (4500 mg), B6 (1000 mg), B12 (5 mg), E (7500 mg), biotin (65 mg), D3 (1,000,000 IU), K3 (2500 mg), folic acid (650 mg), nicotinic acid (10,000 mg), pantothenic acid (6500 mg), plus calcium carbonate as a carrier. Minerals: Co (60 mg), Fe (10 g), I (0.24 g), Se (65 mg), Zn (50 g), Cu (3.0 g), Mn (60 g).^2^ Gross energy (GE, kcal/100g DM) was estimated via NRC (1994) values: lipid (9.44 kcal/g), carbohydrate (4.12 kcal/g), and protein (5.64 kcal/g).^3^ P/E ratio: Protein-to-energy ratio.Experimental diets: D1 (control diet without BAs); diets D2, 3, 4, 5, and 6 contained commercial BAs 0.10, 0.20, 0.30, 0.40, and 0.50 g/kg, respectively.

### Experiment design

This research assessed the impact of nutritional BAs dietary inclusion on growth, nutrient utilization, proximate body structure, immunity, antioxidant activity, liver and intestinal morphology, microbiota, and lipid metabolism gene expression (Adipose triglyceride lipase; ATGL, Hormone-sensitive lipase; HSL, Peroxisome proliferator-activated receptor α; PPARα, Carnitine palmitoyl transferase 1; CPT^−1^, Fatty acid synthase; FAS, Insulin-like growth factor^−II^; Igf^−II^) in *O. niloticus* fingerlings. Monosex Nile tilapia fingerlings (9.0 ± 0.5 g) were sourced from a freshwater farm in Kafr El-Sheikh, Egypt. After a 14-day acclimation, 180 healthy fish were randomly assigned to 18 hapas (0.7 × 0.7 × 1.0 m) in cement raceway ponds at Baltim Research Station, with three replicate groups (10 fish per hapa). Aeration was maintained using air stones and an air pump.

Throughout the experiment, trial meals were administered to the fish manually twice daily, six days a week. The dietary administration level was set at 3% of the total fish biomass during the experimental period until the experiment’s end on day 70. Fish were weighed biweekly to ensure an adequate and balanced feeding regime, and the recorded biomass data were used to adjust feed quantities accordingly.

**Sample collection and analysis**:

### Water Physico-chemical parameters

Water quality was monitored throughout the 70-day experiment. pH, temperature, and dissolved oxygen were determined via a HANNA multiparametric device (HI 9829). Nitrogenous compounds (NH₃, NO₂, NO₃) were analyzed with a DREL 2000 spectrophotometer (HACH) following APHA^25^ guidelines.

The recorded water temperature remained stable throughout the experiment, averaging 28.40 ± 0.07 °C. The pH levels were maintained at an average of 7.63 ± 0.21, while dissolved oxygen concentrations averaged 5.46 ± 0.21 mg/L. The concentrations of nitrogenous compounds were as follows: NH₃ at 0.02 ± 0.001 mg/L, NO₂ at 0.20 ± 0.01 mg/L, and NO₃ at 3.53 ± 0.13 mg/L. These indices remained within the suitable range for Nile tilapia culture, ensuring a suitable aquatic environment for the study.

### Growth metrics and feed efficiency

Cho and Kaushik^26^ formulae were used to calculate feed efficiency factors and fish growth rates for 3 fish each hapa. This includes metrics like energy usage (EU), protein productive value (PPV), specific growth rate (SGR), feed conversion ratio (FCR), and average daily gain (AWG). Assessing nutrient efficiency, overall growth performance of mass weighing, and fish development requires careful attention to each measure by equations: Could you please make these equations image smaller? Many thanks!


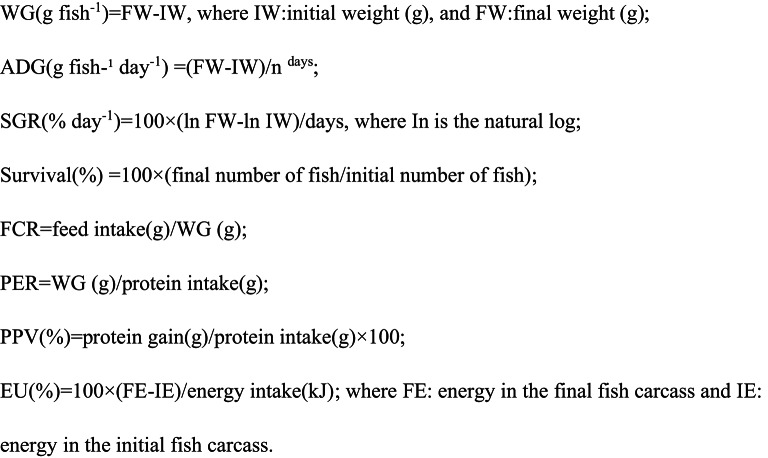


### Chemical and blood analysis of experimental fish

The approximate chemical structure of three trial fish from each hapa, such as moisture, crude protein, crude fat, and ash content, was assessed via^27^ procedures. At the finish of the experiment, three fish from each hapa were stored at −18 °C for carcass structure analysis. Moisture was evaluated by dehydrating samples at 105 °C, percentage of ash by burning at 550 °C, crude lipid by the extraction technique of Soxhlet, and crude protein by the Kjeldahl technique (*N* = 6.25). Dry weight was calculated after dehydration at 55 °C.

For blood analysis, three samples from each hapa were collected after anesthetizing fish with clove oil (5 mL/L)^28^. Blood collected from caudal vessels was divided into deuce sets: one with anticoagulant (0.1 mL sodium citrate) for hematological assessments (White Blood Cell count; WBC, Red Blood Cell count; RBC, hematocrit, hemoglobin, and phagocytic activity) and another without anticoagulant for serum analysis. The serum was centrifugated at 2000 × rpm for ten minutes and saved at −20 °C for biochemical investigation, such as lysozyme activity and immune responses.

Biochemical parameters were analyzed using standard protocols: RBC and Packed Cell Volume; PCV was determined with a Neubauer hemocytometer^29^, hemoglobin concentration via the cyanomethemoglobin method^29,30^, and serum aspartate aminotransferase; AST, alanine aminotransferase; ALT activities, total protein, albumin, and globulin levels were measured following established methods^31^ by using commercial kits “Spinreact, S.A., Gerona, Spain”.

Hematological assessments included WBC counts, leukocyte composition, and Total immunoglobulins; IgM levels were evaluated via assay kits. Serum lysozyme activity was evaluated by the turbidimetric method with *Micrococcus lysodeikticus* (Sigma-Aldrich, USA) suspension, according to^32^, while phagocytic activity and index using the hemocytometer to calculate the number of engulfed yeast (*Candida albicans*) cells by phagocytic cells, and was calculated subsequent equations by^33^; Could you please make these equations image smaller? Many thanks!


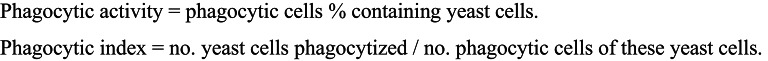


Antioxidant activity was measured using diagnostic kits “MyBioSource Inc., USA”: Superoxide Dismutase activity; SOD activity^34^, catalase; CAT^35^, Malondialdehyde level; MDA^36^, and Total antioxidant Capacity; TAC^37^.

### Histological study

Liver and intestinal tissues of three fish per treatment were collected, dissected, and immersed in 10% buffered formalin for a day, dehydrated, fixed in paraffin, segmented (4–5 μm), dyed with H&E, and examined under an Olympus BX50 microscope^38^.

### Gene expression by real-time quantitative

RT-qPCR was utilized to examine gene expression in 3 samples from each experimental treatment of the liver, peritoneal fat, and muscle to assess the influence of bile acid (BA) addition on lipid metabolism, bile acid synthesis, and protein metabolism. Target genes included ATGL, PPARα, FAS, HSL, CPT^−1^ (lipid metabolism; bile acid synthesis), and Igf^−II^ (protein metabolism). Primer sequences are provided in Table 2.

Total RNA was extracted using RNAiso Plus (TaKaRa), and its quality was verified by gel electrophoresis and spectrophotometry. cDNA was produced from 1 µg RNA utilizing the PrimeScript^®^ RT chemical kit (TaKaRa). RT-qPCR was achieved using a Bio-Rad CFX 96 system with SYBR^®^ Premix Ex Taq™ II. Cycling conditions included an initial denaturation (95 °C, 3 min) shadowed by 38 sequences of denaturation (95 °C, 15 s), annealing (60 °C, 30 s), and delay (65 °C, 5 s). A melting curve analysis confirmed product specificity. Gene expression levels were evaluated via the 2^−ΔΔct^ technique, with *β-actin* as a “housekeeping gene”^39,40^.

**Table 2:** Primer sequences used in real-time quantitative PCR

Gene	Accession number	Direction	Size of primers (5′−3′)	Annealing Temperature °C	Primer efficiency
ATGL	HQ845211	R	GCTCGTACTGAGGCAAATTA	58	2.11
		F	TCGTGCAAGCGTGTATATG		
PPARα	FJ623265	R	ACGTCACCTGGTCATTTAAG	61	2.03
		F	CGCTGAGGTTCGGATATTT		
CPT^−1^	JF728839.1	R	TTAAGGCCCATAGTTCCATTC	56	1.99
		F	GTTACACTGGATGACACAGAG		
Igf^–I I^	EU272149.1	R	CACAGTACATCTCAAGGCGC	60	1.89
		F	CACCCTCTCACTACTGCTGT		
FAS	GQ466046	R	CTCTTCAGCAAGGGAGTTTAG	58	2.15
		F	CCTCAGCTTACAGCAGAATC		
HSL	HQ446238	R	AAGCGCACGTTGACTGG	62	2.02
		F	TGGAACGTTACTGAGTCTGG		

ATGL, adipose triglyceride lipase; PPARα, peroxisome proliferator-activated receptor α; CPT^-1^, carnitine palmitoyl transferase 1; Igf-II insulin-like growth factor-II; FAS, fatty acid synthase; HSL, hormone-sensitive lipase.

### Statistical analysis

The results are displayed as the mean ± the standard deviation (SD). The statistical examination, Duncan’s test (*P* ≤ 0.05), and one-way ANOVA were performed via SPSS 17.0.

## Results

### Growth metrics and survival rate (%)

Table 3 presents the final body weight, TWG, ADG, and SGR % values. The greatest growth performance was in D3, which contained 0.2 g of commercial bile acid (BA) per kg diet. A statistically substantial variance (*P* ≤ 0.05) was detected between all groups. Nile tilapia fingerlings fed the D3 diet, enriched with 0.2 g/kg of BAs, exhibited significantly enhanced growth performance, with TWG of 26.18 g/fish, ADG of 0.37 g/fish/day, and SGR of 1.89%/day. In contrast, fish given the control diet (D1) exhibited markedly lowest values, with a TWG of 12.23 g/fish, an ADG of 0.17 g/fish/day, and an SGR of 1.17%/day. Additionally, the highest (*P* ≤ 0.05) survival rate was noted in the treatment D3, indicating the beneficial effects of bile acid supplementation at this level.

**Table 3:** Impact of diverse levels of commercial bile acid on nile tilapia fingerlings’ growth metrics and survival rate.

Diets ^1^	Body weight (g/fish)		TWG^2^ (g/fish)	ADG^3^ (g/fish/day)	SGR^4^ (%/day)	Survival rate (%)
	Initial	Final				
D1	9.59 ± 0.01	21.82^f^ ± 0.03	12.23^f^ ± 0.20	0.17^f^ ± 0.002	1.17^f^ ± 0.002	95.00^f^ ± 1.00
D2	9.51 ± 0.01	26.30^d^ ± 0.01	16.79^d^ ± 0.21	0.24^d^ ± 0.001	1.45^d^ ± 0.002	96.30^d^ ± 1.10
D3	9.55 ± 0.01	35.73^a^ ± 1.05	26.18^a^ ± 0.56	0.37^a^ ± 0.001	1.89^a^ ± 0.002	98.00^a^ ± 1.10
D4	9.49 ± 0.05	32.37^b^ ± 1.33	22.88^b^ ± 0.21	0.33^b^ ± 0.001	1.75^b^ ± 0.001	97.00^b^ ± 1.12
D5	9.53 ± 0.01	29.89^c^ ± 1.54	20.36^c^ ± 0.18	0.29^c^ ± 0.001	1.63^c^ ± 0.002	96.50^c^ ± 1.15
D6	9.52 ± 0.02	23.72^e^ ± 0.75	14.20^e^ ± 0.20	0.20^e^ ± 0.001	1.30^e^ ± 0.001	96.00^e^ ± 1.00

^1^D1 (control diet without BAs); diets 2, 3, 4, 5 and 6 were containing commercial BAs 0.10, 0.20, 0.30, 0.40,

and 0.50 g/kg diet, respectively.

^2^TWG=total weight gain. ^3^ADG= Average daily gain.^4^SGR= Specific growth rate.

Means followed by different letters in the same column differ significantly (P≤ 0.05), n=9.

### Feed consumption and utilization

Dietary bile acid (BA) supplementation significantly influenced feed consumption and utilization in Nile tilapia fingerlings (Table 4). Fish fed the D3 diet (0.2 g BA/kg) showed the highest feed intake (32.73 g/fish), significantly greater than all other treatments, while the control (D1) had the lowest intake (24.34 g/fish). FCR was most efficient in D3 (1.25), whereas D1 had the poorest FCR (1.99), indicating lower feed utilization efficiency. Similarly, protein efficiency ratio (PER) was highest in D3 (2.63), followed by D4 (2.27) and D5 (2.04), with the lowest recorded in D1 (1.65). Protein productive value (PPV%) also peaked in D3, significantly higher than in D1, D2, and D6. Energy utilization (EU%) exhibited a similar trend, with D3 achieving the highest value (33.28%), outperforming D1 (16.18%), D2 (21.14%), and D6 (19.15%). These results indicate that 0.2 g BA/kg (D3) optimally enhances feed consumption and utilization.

**Table 4:** Impact of diverse concentration of commercial bile acid on feed consumption and efficiency of nile tilapia fingerlings.

Diets^1^	Feed intake (gm/fish)	Feed conversion ratio (FCR)	Protein utilization		Energy utilization (EU %)
			Protein efficiency ratio (PER)	Protein productive value (PPV%)	
D1	24.34^e^ ± 0.02	1.99^a^ ± 0.002	1.65^e^ ± 0.01	28.05^f^ ± 0.15	16.18^f^ ± 0.02
D2	28.54^c^ ± 0.02	1.70^b^ ± 0.002	1.93^c^ ± 0.01	35.26^d^ ± 0.18	21.14^d^ ± 0.11
D3	32.73^b^ ± 0.03	1.25^e^ ± 0.003	2.63^a^ ± 0.01	52.48^a^ ± 0.14	33.28^a^ ± 0.03
D4	33.17^a^ ± 0.02	1.45^d^ ± 0.005	2.27^b^ ± 0.01	42.32^b^ ± 0.25	26.35^b^ ± 0.15
D5	32.78^b^ ± 0.14	1.61^c^ ± 0.001	2.04^c^ ± 0.01	37.08^c^ ± 0.23	22.52^c^ ± 0.11
D6	24.84^d^ ± 0.02	1.75^b^ ± 0.002	1.88^d^ ± 0.01	32.77^e^ ± 0.11	19.15^e^ ± 0.04

^1^D1 (control diet without BAs); diets 2, 3, 4, 5, and 6 were containing commercial BAs 0.10, 0.20, 0.30, 0.40,and 0.50 g/kg diet, respectively. Means followed by different letters in the same column differ significantly (P≤ 0.05), n=9.

### Carcass composition

After the trial, the whole-body carcass proximate structure was examined to assess dry matter (DM), crude protein (CP), ether extract (EE), ash, and energy content, as shown in Table 5. DM content was insignificant (*P* ≤ 0.05) among all treatments. Nevertheless, substantial differences (*P* ≤ 0.05) were detected in CP, EE, ash, and energy content. Fish fed the D3 diet (0.2 g BA/kg) exhibited the highest CP content (64.50%), significantly surpassing other groups. Conversely, the fish given the D1 and D6 diets exhibited the lowest EE content, implying enhanced lipid metabolism. Ash content was highest in the control group (D1) at 20.01%, whereas D3 (0.2 g BA/kg) had the lowest value (10.12%). Energy content also varied significantly, with D1 showing the lowest value (533.34 Kcal/100 g) and D3 the highest (604.27 Kcal/100 g). These results indicate that dietary BA improves protein deposition, optimizes fat metabolism, and affects energy utilization in *O. niloticus* fingerlings.

**Table 5:** Impact of various concentrations of commercial bile acid on the whole body composition of *O. niloticus* fingerlings.

Diets ^1^	Dry Matter %	% On a dry matter basis			Energy Content (Kcal/100 g diet)
		Crude protein	Ether extract	Ash	
At the start:	25.15 ± 0.13	55.57 ± 0.11	27.56 ± 0.12	16.87 ± 0.15	574.41 ± 0.25
At the end:					
D1	26.75^d^ ± 1.18	58.57^e^ ± 0.22	21.42^d^ ± 0.23	20.01^a^ ± 0.09	533.34^e^ ± 0.32
D2	27.37^c^ ± 1.02	61.03^c^ ± 0.19	22.58^c^ ± 0.20	16.39^c^ ± 0.13	558.20^c^ ± 0.34
D3	28.48^a^ ± 1.11	64.50^a^ ± 0.33	25.38^a^ ± 0.04	10.12^f^ ± 0.17	604.27^a^ ± 0.28
D4	27.92^b^ ± 1.07	61.97^b^ ± 0.31	23.82^b^ ± 0.16	14.21^e^ ± 0.31	575.23^b^ ± 0.24
D5	27.50^c^ ± 1.11	61.22^c^ ± 0.27	22.76^c^ ± 0.02	16.02^d^ ± 0.08	560.98^c^ ± 0.34
D6	26.95^d^ ± 1.27	59.60^d^ ± 0.18	21.67^d^ ± 0.07	18.73^b^ ± 0.22	541.52^d^ ± 0.35

^1^D1 (control diet without BAs); diets 2, 3, 4, 5, and 6 were containing commercial BAs 0.10, 0.20, 0.30, 0.40, and 0.50 g/kg diet, respectively.

Means followed by different letters in the same column differ significantly (P≤ 0.05), n=9 at the start and the end of experimental fish

### Biochemical parameters

**Fig. 1:**
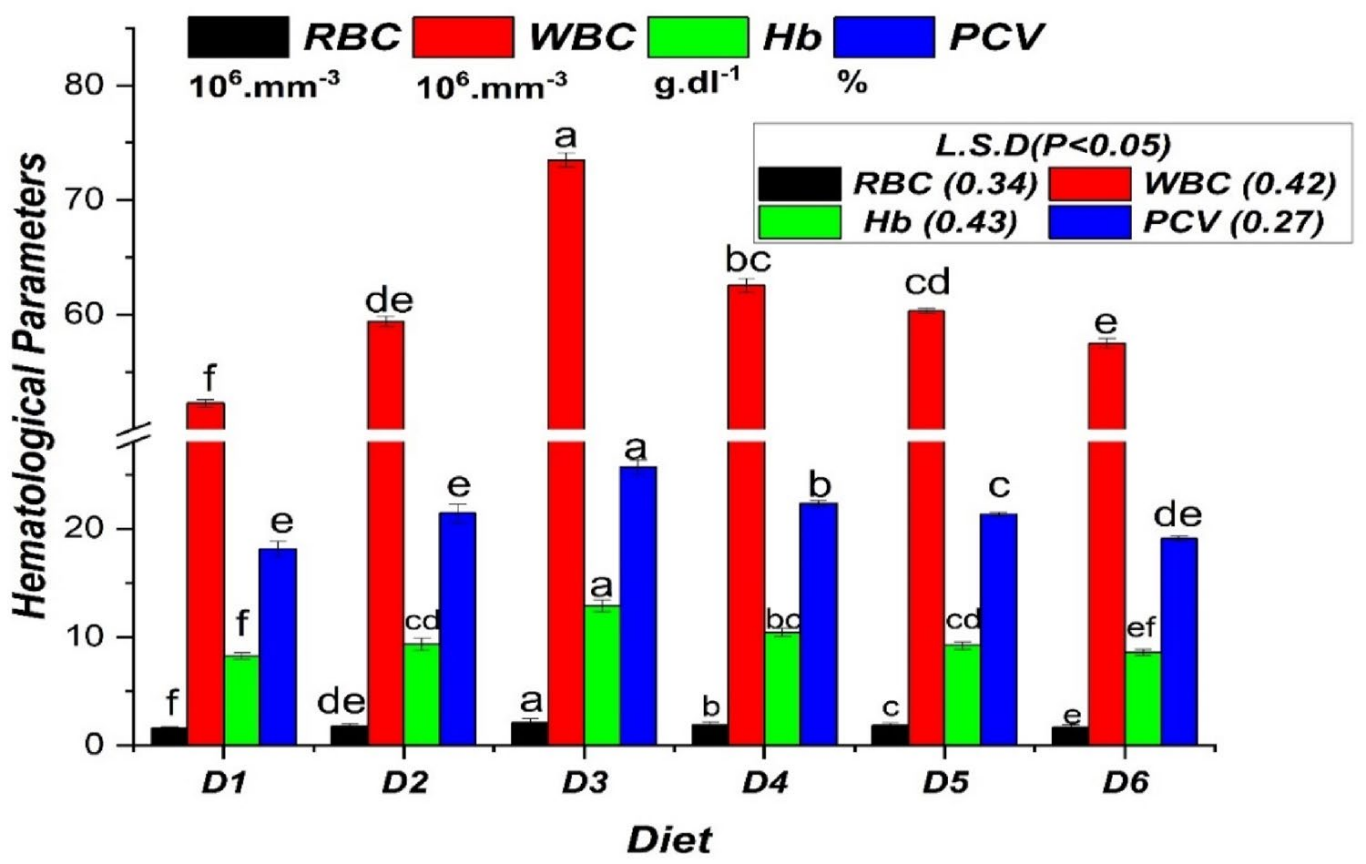
Hematological parameters of Nile tilapia (*O. niloticus*) fingerlings fed diets supplemented with graded levels of commercial bile acids (BAs). Grouped bars represent the mean values (*n* = 9) ± standard error (SE) for Red Blood Cell count (RBC, 10⁶/mm³), White Blood Cell count (WBC, 10³/mm³), Hemoglobin concentration (Hb, g/dl), and Packed Cell Volume (PCV, %). Within each parameter group (indicated by color), bars topped with different letters show statistically significant differences between dietary groups (*P* ≤ 0.05).

Hematological indices (RBCs, Hb, and PCV) of *O. niloticus* fish fed varied levels of commercial Bile acid are presented in Fig. 1. The highest value of the hematological indices (RBCs, Hb, and PCV) was found for fish fed on D3, which contained 0.2 g commercial Bile acid (BA)/kg diet, and there was a substantial difference (*P* ≤ 0.05) amongst all treatments. The erythrogram revealed that RBCs, (2.08 10^6^/mm^3^); total WBCs, (73.47 10^3^/mm^3^); Hb, (12.89 g/dl) and PCV, (25.67%), respectively, were significantly highest treatment.

### Hematological indices

The hematological indices of *O. niloticus* fed diets with changeable concentrations of commercial bile acid are summarized in Table 6. Fish on the D3 diet (0.2 g/kg bile acid) showed the highest lymphocyte values, with significant variations among all groups (*P* ≤ 0.05). Basophil, eosinophil, and neutrophil counts decreased with bile acid levels from D2 to D6 (0.1–0.5 g/kg). Monocyte counts D3 have differed significantly among treatments.

Biochemical indices showed significantly higher total protein (3.93 and 3.82 g/dl) in the D3 and D4 groups, while serum albumin and globulin increased with bile acid supplementation. However, total globulin levels declined as bile acid inclusion rose. AST and ALT values were significantly greater in the control (D1) than in bile acid-supplemented groups.

For immune parameters, D3 and D4 diets significantly enhanced phagocytic activity (50.21and 44.73), phagocytic index (9.29 and 8.75), lysozyme (0.39 and 0.36), and total immunoglobulins (2.29 and 1.88). However, as bile acid levels increased beyond D6, immune markers declined. These findings suggest moderate bile acid supplementation enhances immune responses, with optimal benefits at 0.1–0.3 g/kg.

**Table 6:** Hematological and biochemical responses of nile tilapia fingerlings fed commercial bile acid supplementation.

Diet^1^		D1	D2	D3	D4	D5	D6
Differential leucocytic counts Parameters	Lymphocytes	55.18^f^ ± 0.43	63.91^d^ ± 0.57	72.44^a^ ± 0.29	69.45^b^ ± 0.67	66.33^cd^ ± 0.55	61.11^e^ ± 0.44
	Monocytes	2.65^a^ ± 0.04	2.08^a^ ± 0.04	1.89^b^ ± 0.03	2.07^a^ ± 0.07	2.45^a^ ± 0.07	2.53^a^ ± 0.08
	Basophils	11.37^a^ ± 0.47	10.78^b^ ± 0.54	9.88^c^ ± 0.74	8.02^d^ ± 0.47	7.89^e^ ± 0.59	8.33^d^ ± 0.81
	Eosinophil	11.75^a^ ± 0.83	10.67^b^ ± 0.55	10.07^b^ ± 0.34	8.13^d^ ± 0.29	9.08^c^ ± 0.63	9.03^c^ ± 0.51
	Neutrophils	23.31^a^ ± 1.88	14.71^c^ ± 1.62	10.22^d^ ± 1.33	13.17^cd^ ± 1.67	17.81^b^ ± 1.55	22.07^ab^ ± 1.53
Differential leucocytic counts Parameters	Lymphocytes	55.18^f^ ± 0.43	63.91^d^ ± 0.57	72.44^a^ ± 0.29	69.45^b^ ± 0.67	66.33^cd^ ± 0.55	61.11^e^ ± 0.44
	Monocytes	2.65^a^ ± 0.04	2.08^a^ ± 0.04	1.89^b^ ± 0.03	2.07^a^ ± 0.07	2.45^a^ ± 0.07	2.53^a^ ± 0.08
	Basophils	11.37^a^ ± 0.47	10.78^b^ ± 0.54	9.88^c^ ± 0.74	8.02^d^ ± 0.47	7.89^e^ ± 0.59	8.33^d^ ± 0.81
	Eosinophil	11.75^a^ ± 0.83	10.67^b^ ± 0.55	10.07^b^ ± 0.34	8.13^d^ ± 0.29	9.08^c^ ± 0.63	9.03^c^ ± 0.51
Serum biochemical parameters	TP g/dl	3.24^e^ ± 0.07	3.33^d^ ± 0.04	3.93^a^ ± 0.05	3.82^b^ ± 0.01	3.65^c^ ± 0.07	3.32^d^ ± 0.02
	Alb g/dl	1.91^c^ ± 0.05	1.89^cd^ ± 0.02	1.94^b^ ± 0.02	2.08^a^ ± 0.01	2.05^a^ ± 0.05	2.07^a^ ± 0.02
	Glob g/dl	1.33^e^ ± 0.06	1.44^d^ ± 0.02	1.99^a^ ± 0.03	1.74^b^ ± 0.04	1.61^c^ ± 0.04	1.25^f^ ± 0.02
	Alb/Glob	1.55^b^ ± 0.03	1.39^c^ ± 0.02	0.86^e^ ± 0.03	1.16^d^ ± 0.02	1.37^c^ ± 0.03	1.66^a^ ± 0.03
	S. AST IU/L	23.48^a^ ± 0.19	21.39^b^ ± 0.27	18.44^c^ ± 0.55	19.29^c^ ± 1.12	17.51^d^ ± 0.37	21.62^b^ ± 0.34
	S. ALT IU/L	28.59^a^ ± 0.44	27.29^ab^ ± 0.39	21.79^e^ ± 0.39	23.67^d^ ± 0.29	24.57^c^ ± 0.72	25.19^c^ ± 0.33
Immune status parameters	PA	23.79^f^ ± 1.42	33.62^e^ ± 1.47	50.21^a^ ± 1.84	44.73^b^ ± 1.24	41.38^c^ ± 1.61	38.51^d^ ± 1.34
	PI	3.39^f^ ± 0.16	6.17^e^ ± 0.23	9.29^a^ ± 0.38	8.75^b^ ± 0.22	7.69^cd^ ± 0.41	6.88^de^ ± 0.23
	Lys U/ml	0.22^f^ ± 0.03	0.26^e^ ± 0.04	0.39^a^ ± 0.03	0.36^b^ ± 0.04	0.34^c^ ± 0.05	0.33^d^ ± 0.04
	TI g/dL	1.25^f^ ± 0.07	1.62^e^ ± 0.03	2.29^a^ ± 0.06	1.88^bc^ ± 0.05	1.83^c^ ± 0.04	1.65^de^ ± 0.06

^1^D1 (control diet without BAs); diets 2, 3, 4, 5, and 6 were containing commercial BAs 0.10, 0.20, 0.30, 0.40, and 0.50 g/kg diet, respectively.

Means followed by different letters in the same raws differ significantly (P≤ 0.05), n=9.

TP = Total protein, Alb = Albumin, Glob = Globulin, Alb/ Glob = Albumin/ Globulin, AST = aspartate aminotransferase, ALT = alanine aminotransferase, PA = Phagocytic activity, PI = Phagocytic index, Lys = Lysozyme, TI = Total immunoglobulins.

### Biomarker variables for antioxidant and oxidative stress

**Fig. 2:**
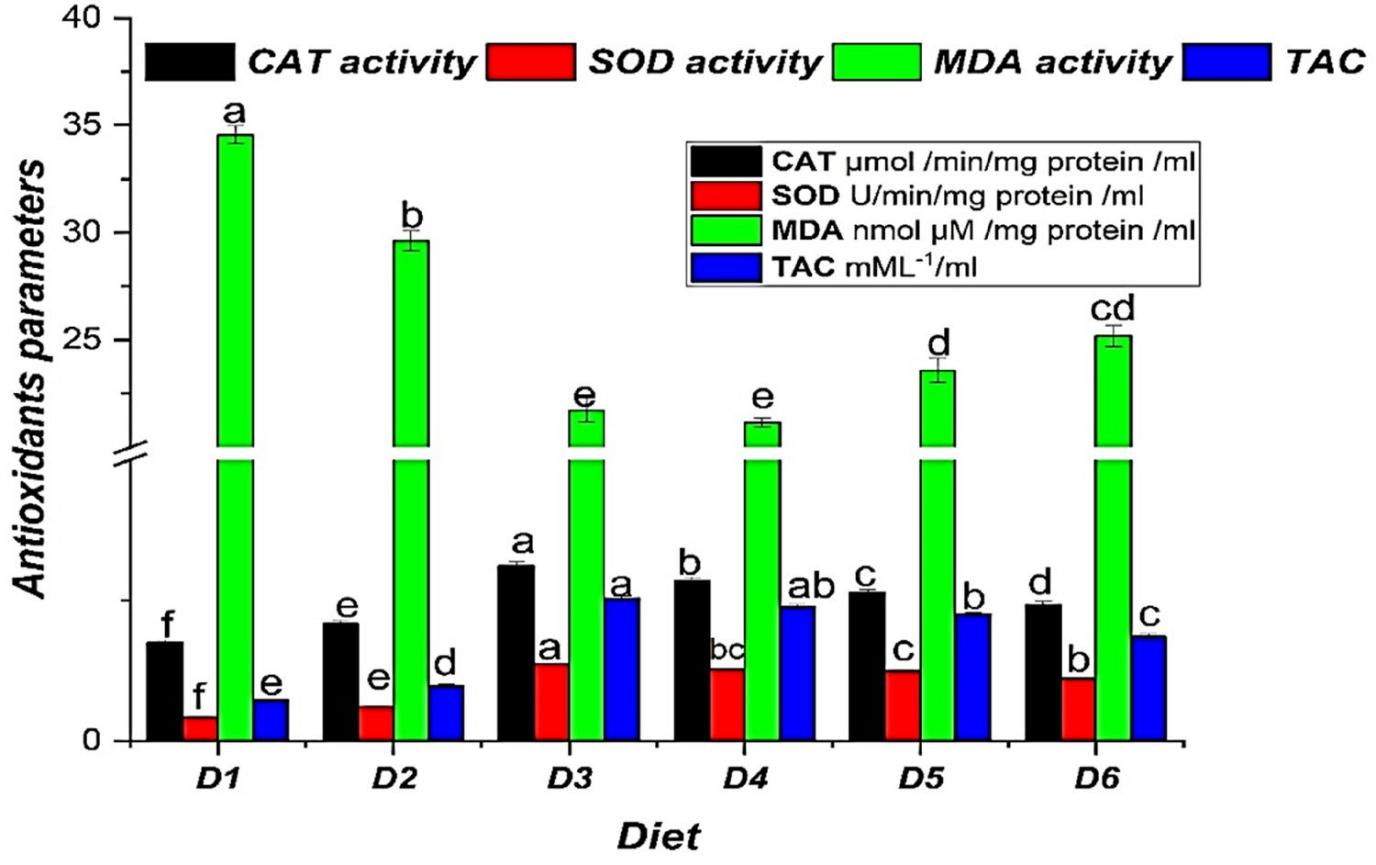
Antioxidant parameters in Nile tilapia (*O. niloticus*) fingerlings fed diets supplemented with graded levels of commercial bile acids (BAs). Bars represent the mean (*n* = 9) ± standard error (SE) for Catalase activity (CAT; µmol/min/mg protein/ml), Superoxide Dismutase activity (SOD; U/min/mg protein/ml), Malondialdehyde level (MDA; nmol µM/mg protein/ml), and Total Antioxidant Capacity (TAC; mML^−1^/ml). Within each measured parameter, bars topped with different letters indicate statistically significant differences between dietary groups (*P* ≤ 0.05).

Antioxidant and oxidative stress biomarkers parameters: (CAT: catalyze activity, SOD: Superoxide dismutase activity, MDA: Malondialdehyde activity, and TAC: Total antioxidants) of Nile tilapia *O. niloticus* fish-fed commercial bile acid are presented in Fig. 2. The highest value of the CAT activity, SOD activity, and TAC values were detected for fish fed on D3, which contained 0.2 g commercial Bile acids/kg diet, and there was significance (*P* ≤ 0.05) between all treatments. Data revealed that CAT activity, SOD activity, and TAC were substantially higher in the D3 and D4 diets related to the other diets. In contrast, MDA activity values decrease with rising concentration levels of commercial BA from D2 to D6 (0.1 to 0.5 g/kg diet).

### Histomorphometric examination of the Gastrointestinal tract and liver

After dietary bile salt supplementation, the histological examination of *O. niloticus* intestines revealed notable morphological differences among treatment groups. The control group (D1), which received no bile acid supplementation, exhibited a typical intestinal wall with well-formed villi, a limited number of goblet cells, and a typical structure, including columnar epithelium, an organized submucosa, and typical absorptive vacuoles, with minor cellular infiltration in the distal intestine.

**Fig. 3:**
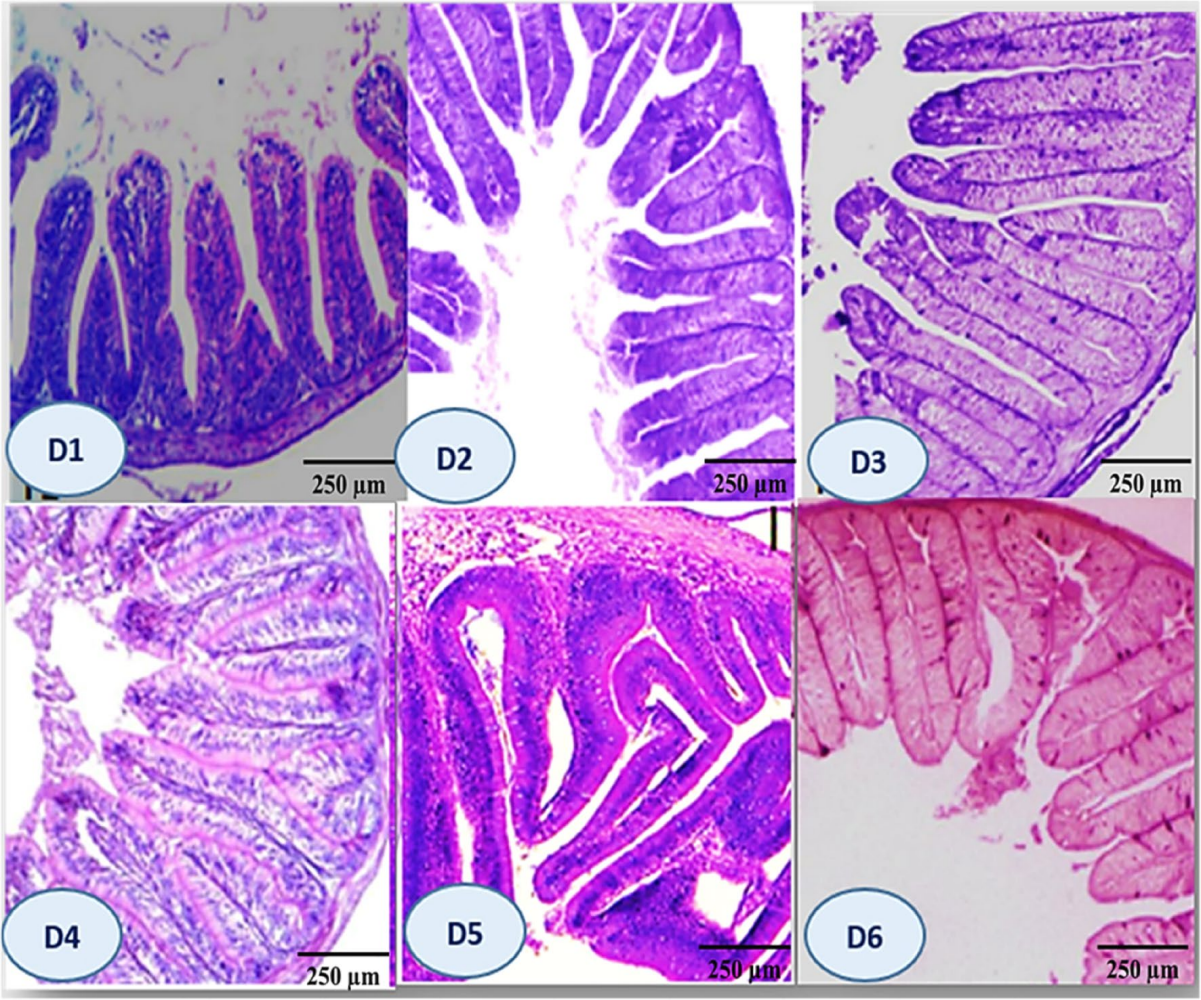
Following 70 days of feeding with different concentrations of commercial bile acids (BA), photomicrographs of the Nile tilapia’s (*O. niloticus*) gastrointestinal tract showed substantial histological changes amongst all treatments (H&E staining, 250× magnification): D1 (Control) had a typical unaltered intestinal wall including well-organized villi, a small count of goblet cells in the mucosa, a well-defined columnar epithelium, submucosa, and standard amounts of absorptive vacuoles. D2: A small number of goblet cells and slightly formed villi were shown, with a modest height rise. D3 demonstrated notable improvements, including augmented villus height, breadth, and crypt depth, enhanced mucosal folds, increased goblet cells in the lamina propria, and heightened levels of absorptive vacuoles. D4: Showed notable improvement in villus length and arrangement, with well-maintained villous architecture and no epithelial degeneration. D5 demonstrated a moderate increase in villus length and structure, preserving near-normal architecture and epithelial integrity. D6 exhibited standard villus height, characterized by significant villus fusion, broad villus tips, decreased goblet cells, mild lymphocytic infiltration, and limited mucosal folds. Figure 4 B: D1 (control diet without BAs); diets 2, 3, 4, 5, and 6 enriched with commercial BAs 0.10, 0.20, 0.30, 0.40, and 0.50 g/kg diet, respectively, n=3.

**Fig. 4:**
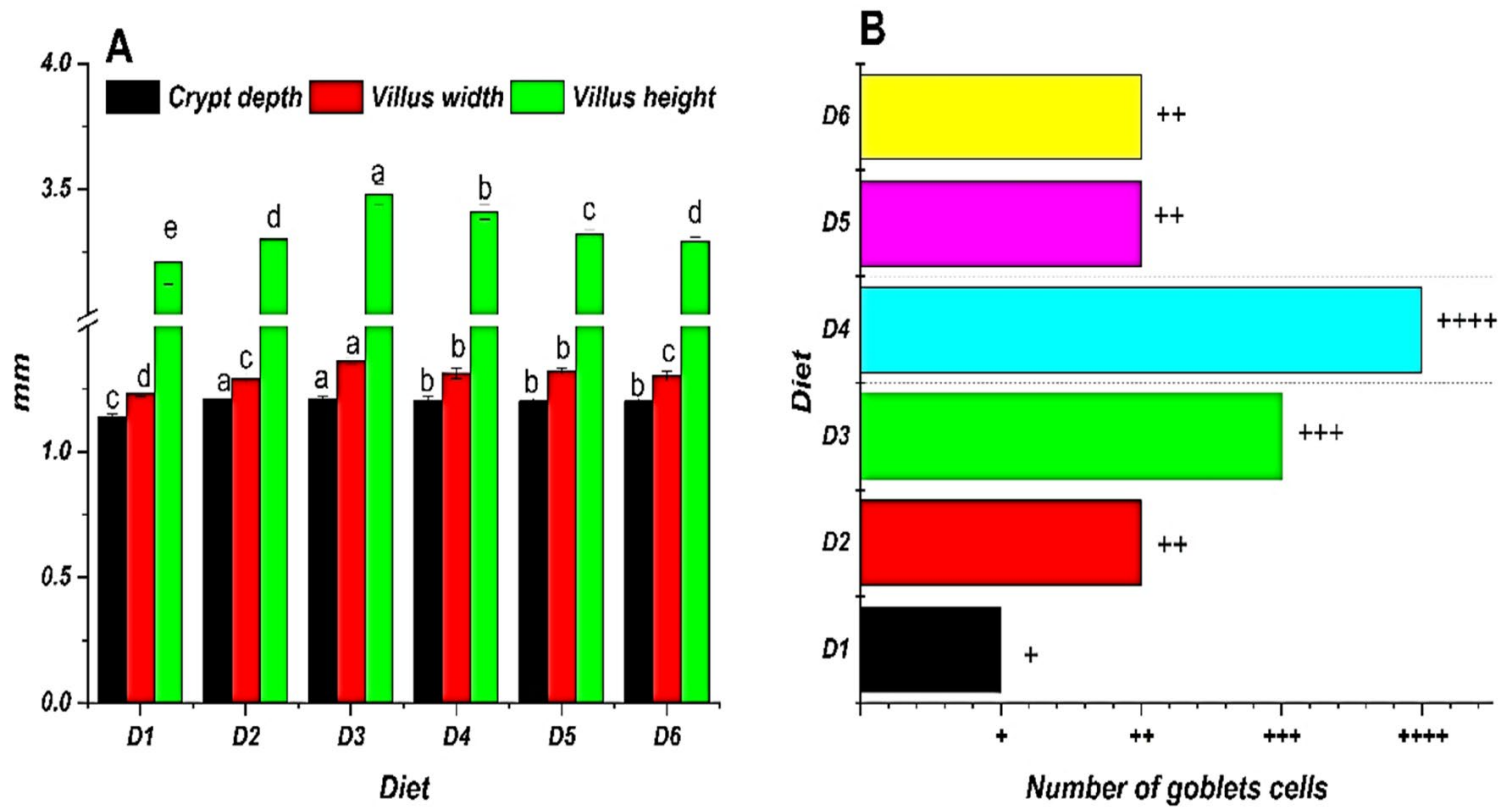
Morphometric analysis of the gastrointestinal tract in Nile tilapia (*O. niloticus*) fingerlings fed diets supplemented with graded levels of commercial bile acids (BAs). **(A)** Mean (± standard error, SE; n=3) crypt depth, villus width, and villus height in millimeters; within each measured parameter, bars topped with different letters indicate statistically significant differences between dietary groups (P≤ 0.05). **(B)** Qualitative representation of goblet cell abundance, indicated by '+' symbols (+, ++, +++, ++++). For statistical comparisons (P<0.05) between dietary groups for each parameter shown in the figure. Diets included a control (D1, 0 g/kg BA) and treatments supplemented with 0.10 (D2), 0.20 (D3), 0.30 (D4), 0.40 (D5), and 0.50 (D6) g/kg BA.

After 70 days of feeding, fish in the D3 group (0.2 g/kg bile acid) displayed the highest villus width, height, and crypt depth, along with increased mucosal folds and goblet cells in the lamina propria. Higher absorptive vacuoles and more cellular infiltration in the distal intestine were observed in D3 compared to D1 (Fig. 3). These structural changes are further supported by the morphometric analysis of the gastrointestinal tract (Fig. 4A).

**Fig. 5:**
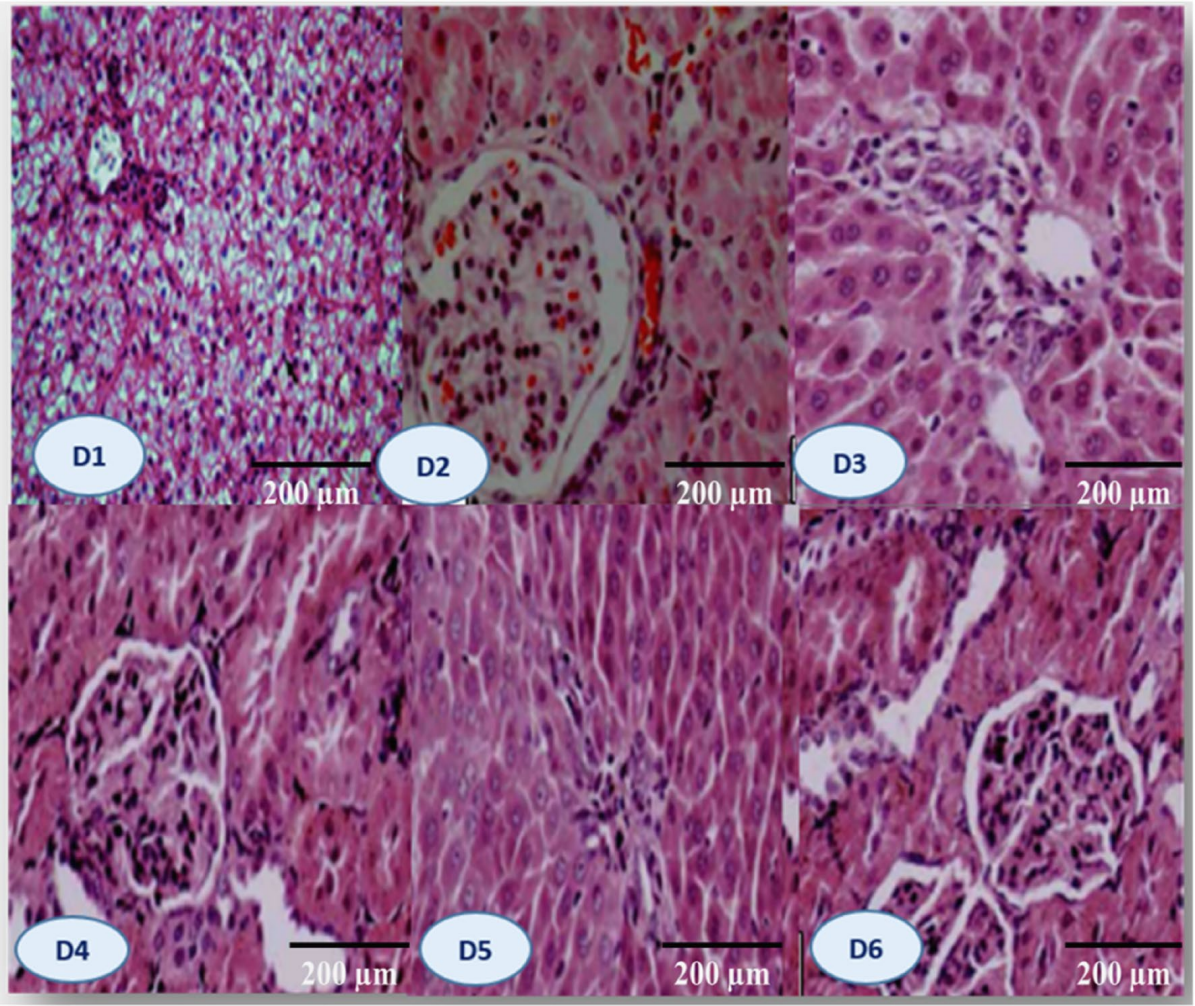
The liver microscopic structure of Nile tilapia feeding with different levels of commercial BA shows round polygonal hepatocytes organized in cord-like formations, constrained on one side by hepatic capillaries or sinusoids. At the core of each cord are narrow bile canaliculi located next to the hepatocytes, along with the pancreatic portion that encircles the afferent vein (D1 control). D2: shows subcapsular leukocytic infiltration and marked lytic and cavitation within the hepatic tissues. D3: showing slight cloudy swelling of hepatocytes and few pyknotic nuclei. D4: demonstrates focal to diffuse vacuolar degeneration of hepatocytes. The vacuoles exhibited severity and diffuseness, accompanied by focal leucocytic infiltration. D5 exhibits diffuse deterioration of hepatocytes, focal necrosis, leukocytic infiltration, and fat cell deposits in the central vein. D6 demonstrates severe deterioration with focal necrosis characterized by pyknotic nuclei, fat cell deposits, alterations in the typical liver architecture, and parenchymal fibrosis with fat deposits (H&E X 200). D1 (control diet without BAs); diets 2, 3, 4, 5, and 6 enriched with commercial BAs 0.10, 0.20, 0.30, 0.40, and 0.50 g/kg diet, respectively, n=3.

Liver histology varied significantly across treatments. In the control (D1), the liver exhibited a typical parenchymal structure, intact hepatoportal veins, and well-defined sinusoids, with hepatocytes containing cytoplasmic vacuoles. In the D3 group, mild hepatocyte erosion and fat deposition around the central vein were observed. The D4 group exhibited more pronounced fat accumulation, disrupting typical liver architecture and initiating parenchymal fibrosis. The most severe changes were found in D6, where extensive fat accumulation led to significant structural distortion of hepatic tissues (Fig. 5).

### Expression genes of lipid metabolic in the liver, peritoneal fat, and muscular tissues

**Fig. 6:**
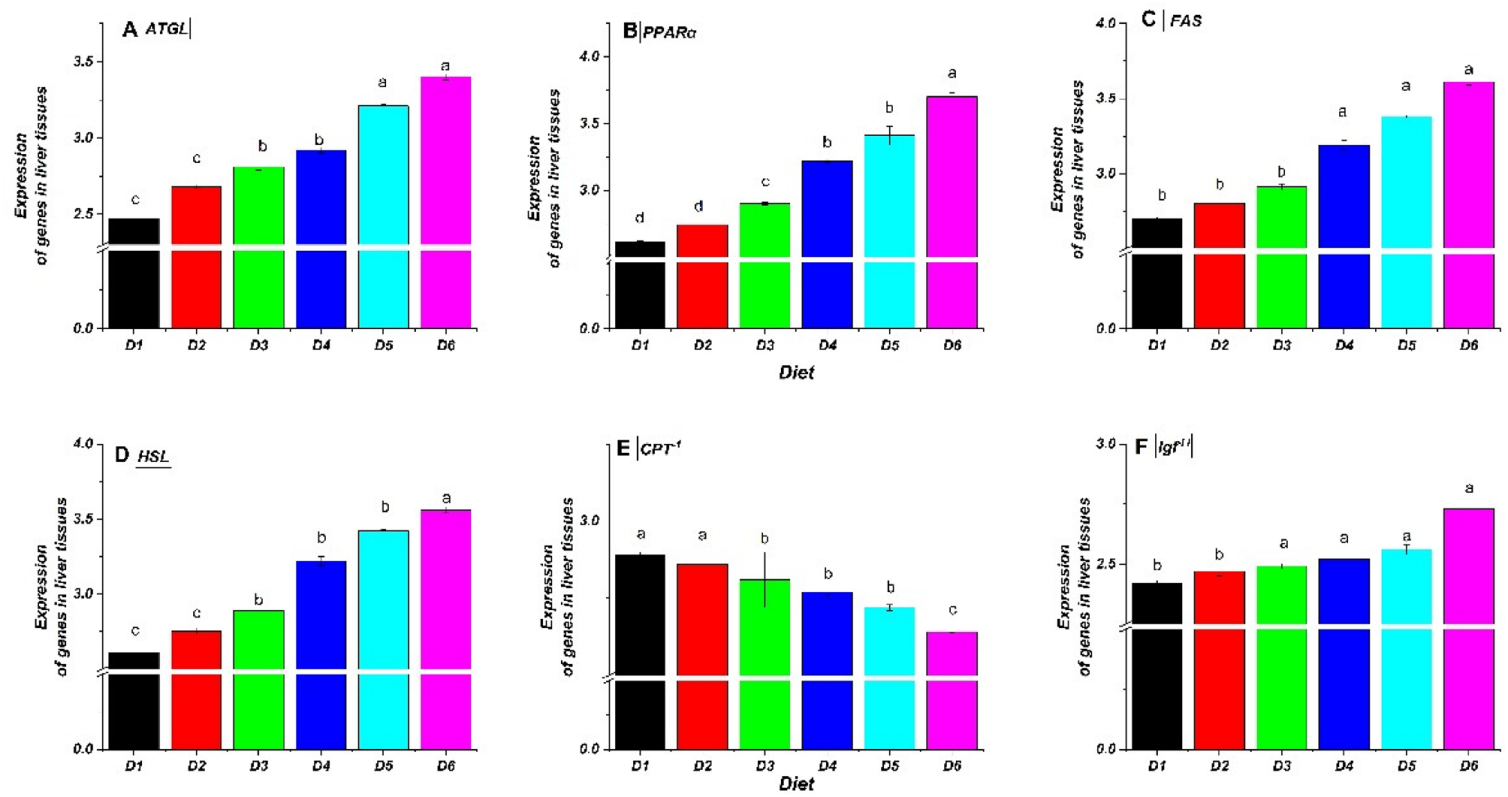
Relative expression of fatty acid metabolism-related genes in the liver tissues of Nile tilapia (*O. niloticus*) fingerlings fed diets supplemented with graded levels of commercial bile acids (BAs). The relative mRNA expression for (**A**) ATGL, (**B**) HSL, (**C**) PPARα, (**D**) CPT^−1^, (**E**) FAS, and (**F**) Igf^−II^. Values are presented as mean ± standard error (SE), *n* = 3 replicates per diet. Within each panel, bars with different letters indicate statistically significant differences (*P* ≤ 0.05). Gene abbreviations: ATGL, adipose triglyceride lipase; PPARα, peroxisome proliferator-activated receptor α; CPT^−1^, carnitine palmitoyl transferase-1; FAS, fatty acid synthase; HSL, hormone-sensitive lipase; Igf^−II^, insulin-like growth factor-II. Diets included a control (D1, 0 g/kg BA) and treatments supplemented with 0.10 (D2), 0.20 (D3), 0.30 (D4), 0.40 (D5), and 0.50 (D6) g/kg BA.

**Fig. 7:**
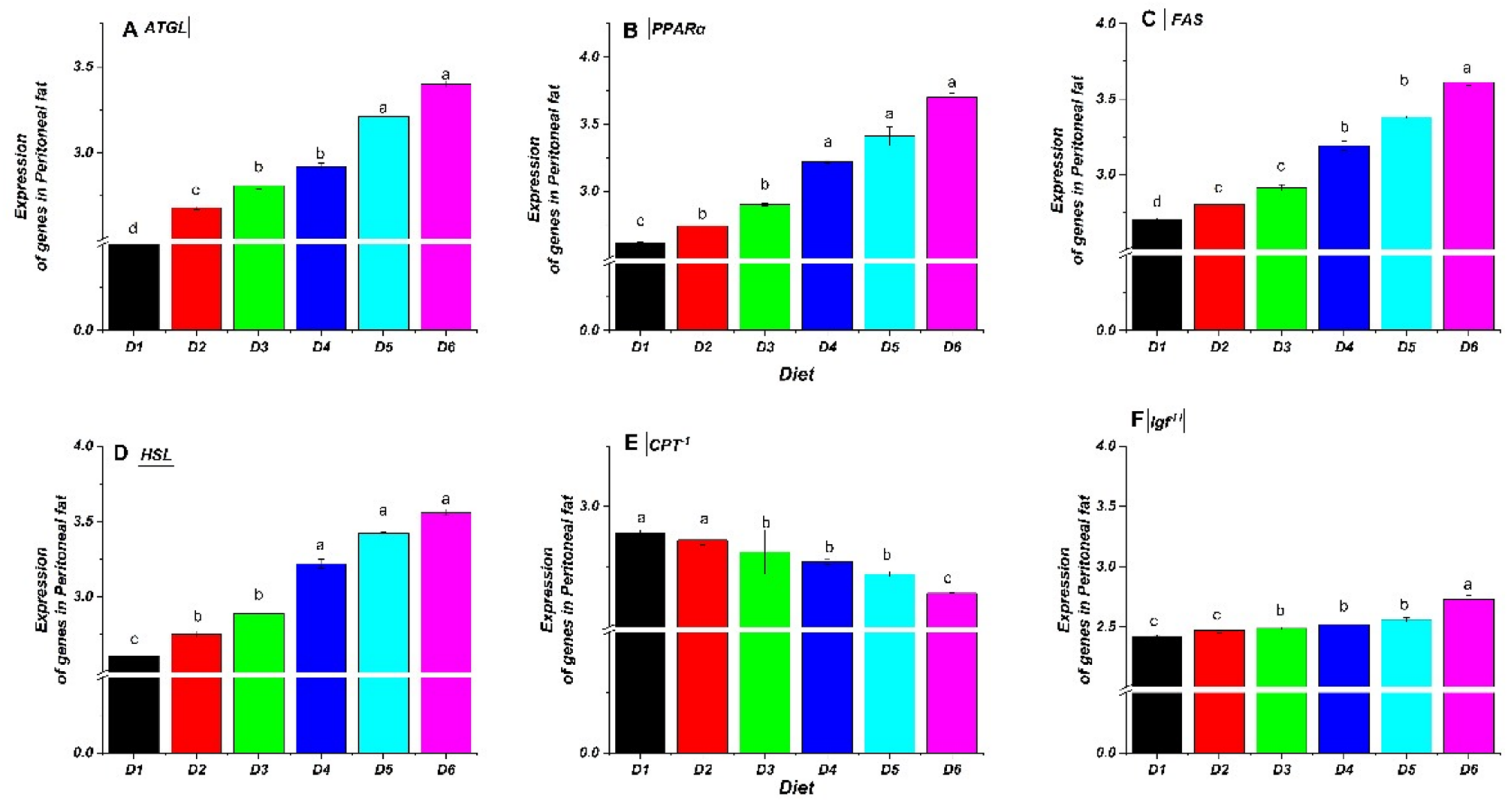
Relative expression of fatty acid metabolism-related genes in the peritoneal fat of Nile tilapia (*O. niloticus*) fingerlings fed diets supplemented with graded levels of commercial bile acids (BAs). The relative mRNA expression for (**A**) ATGL, (**B**) HSL, (**C**) PPARα, (**D**) CPT^−1^, (E) FAS, and (F) Igf^−II^. Values are presented as mean ± standard error (SE), *n* = 3 replicates per diet. Within each panel, bars with different letters indicate statistically significant differences (*P* ≤ 0.05). Gene abbreviations: ATGL, adipose triglyceride lipase; PPARα, peroxisome proliferator-activated receptor α; CPT^−1^, carnitine palmitoyl transferase- 1; FAS, fatty acid synthase; HSL, hormone-sensitive lipase; Igf^−II^, insulin-like growth factor-II. Diets included a control (D1, 0 g/kg BA) and treatments supplemented with 0.10 (D2), 0.20 (D3), 0.30 (D4), 0.40 (D5), and 0.50 (D6) g/kg BA.

**Fig. 8:**
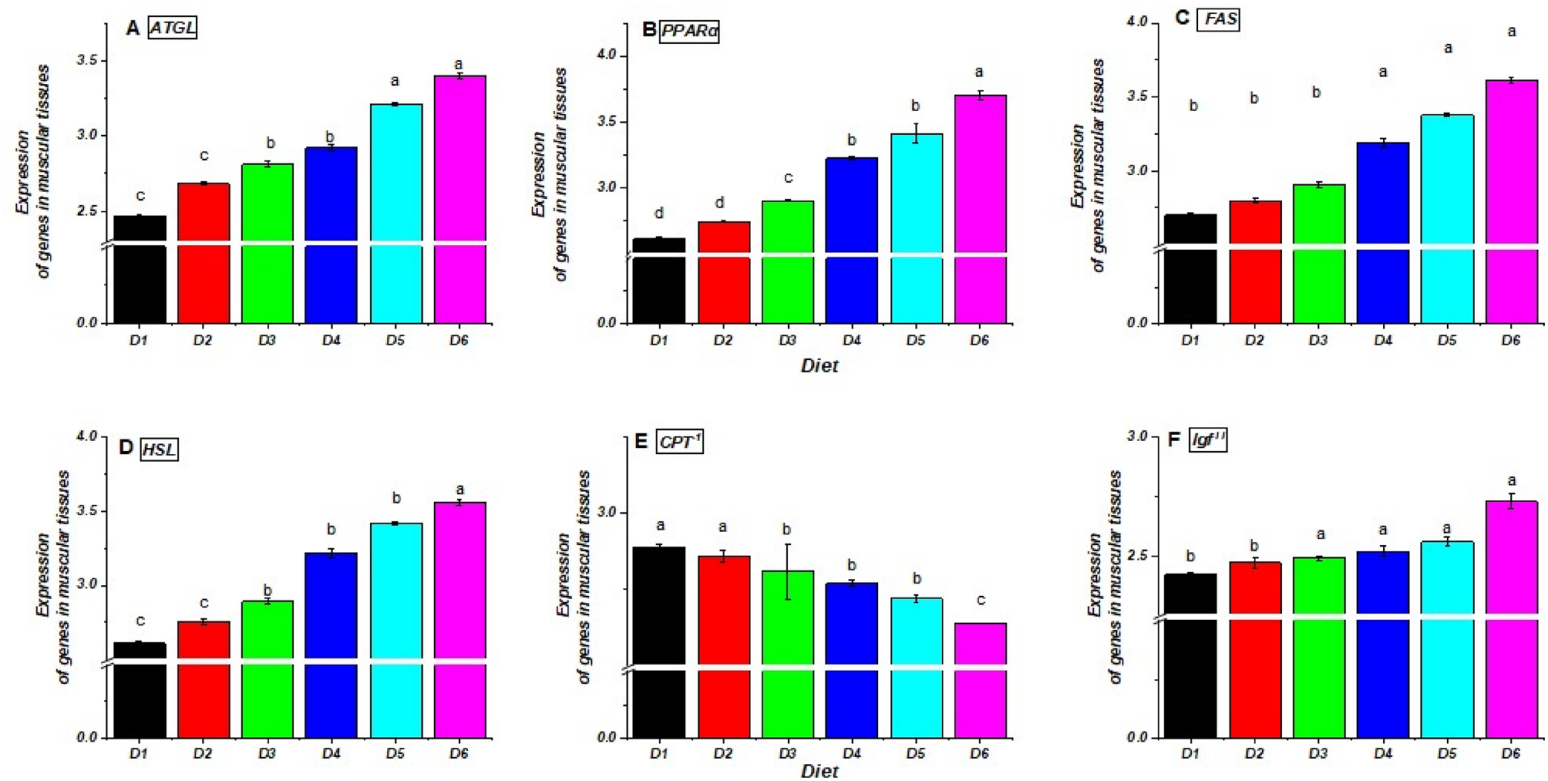
Relative expression of fatty acid metabolism-related genes in the muscular tissues of Nile tilapia (*O. niloticus*) fingerlings fed diets supplemented with graded levels of commercial bile acids (BAs). The relative mRNA expression for (**A**) ATGL, (**B**) PPARα, (**C**) FAS, (**D**) HSL, (**E**) CPT^−1^, and (F) Igf^−II^. Values are presented as mean ± standard error (SE), *n* = 3 replicates per diet. Within each panel, bars with different letters indicate statistically significant differences (*P* ≤ 0.05). Gene abbreviations: ATGL, adipose triglyceride lipase; PPARα, peroxisome proliferator-activated receptor α; CPT^−1^, carnitine palmitoyl transferase- 1; FAS, fatty acid synthase; HSL, hormone-sensitive lipase; Igf^−II^, insulin-like growth factor-II. Diets included a control (D1, 0 g/kg BA) and treatments supplemented with 0.10 (D2), 0.20 (D3), 0.30 (D4), 0.40 (D5) and 0.50 (D6) g/kg BA.

Supplementing the diet of *O. niloticus* fingerlings with bile acid (BA) changed the expression of genes involved in fatty acid metabolics, such as adipose triglyceride lipase (ATGL), carnitine palmitoyl transferase 1 (CPT^−1^), fatty acid synthase (FAS), hormone-sensitive lipase (HSL), peroxisome proliferator-activated receptor α (PPARα), and insulin-like growth factor- II (IGF^−II^). Figures 6 and 7, and 8 illustrate significant modulation of ATGL, FAS, HSL, PPARα, CPT^−1,^ and IGF^−II^ by BA treatments. In liver tissue (Fig. 6), lipolysis-related genes (ATGL, HSL, PPARα) were upregulated in BA-fed groups (D2–D6), with the highest levels in D5 and D6 (0.4 and 0.5 g/kg BA). Conversely, CPT^−1^ and IGF^−II^ expression declined, particularly when the BA dose was increased. In peritoneal fat (Fig. 7), ATGL, FAS, HSL, and PPARα were significantly elevated in BA-treated groups, with D5 and D6 showing the most substantial effects. CPT^−1^ decreased with higher BA levels, mirroring liver results. IGF^−II^ was also lowest in D6. In muscle tissue (Fig. 8), ATGL, FAS, and HSL increased in BA-fed groups, peaking in D5 and D6, while CPT^−1^ and IGF^−II^ declined, consistent with liver and fat tissues. Overall, BA supplementation enhanced lipolysis-related gene expression while downregulating CPT^−1^ and IGF^−II^, indicating potential inhibition of fatty acid oxidation and protein synthesis at higher BA levels. Alterations were statistically significant (*P* ≤ 0.05).

## Discussion

Bile acids possess an amphipathic structure, allowing them to function as detergents that enhance feed digestion and emulsification. They accomplish this by increasing the proteolytic degradation of food proteins and encouraging the production of fat micelles^41^. Improved growth performance in fish generally indicates optimal environmental conditions and adequate nutritional availability^42^. The current investigation includes graded levels of commercial BA in *O. niloticus* diets, significantly enhancing growth performance and feed efficiency. Our findings demonstrated that BA insertion improved the growth of *O. niloticus* fed extruded floating diets containing increasing concentrations of BA. Similar growth-promoting impacts of dietary BA have been concluded in other aquatic animals, such as turbot (*Scophthalmus maximus*)^20^, European seabass (*Dicentrarchus labrax*)^43^, and Atlantic salmon (*Salmo salar L.*)^44^. These results suggest that the positive impact of BA on growth might be due to improved nutrient utilization and digestive enzyme secretions in the intestine.

Additionally, BA supplementation stimulated feed intake, which probably improved growth performance. Bile acids act as chemical signals that influence feeding behavior, potentially due to the very reactive of fish olfactory neurons to these compounds^45,46^. Previous research has indicated that dietary BA supplementation boosts growth and digestive enzyme activity in Japanese eels (*Anguilla japonica*)^47^. Furthermore, numerous studies have highlighted the broader benefits of BA supplementation in aquaculture, including improvements in feed conversion ratio, hepatic performance, digestive enzyme production, and mitigating stressful situations^40,48^. In line with prior research, the current investigation observed that BA inclusion in juvenile Nile tilapia diets substantially enhanced feed efficiency metrics compared to fish-fed control diets. These findings align with earlier studies demonstrating BA-induced growth enhancement in species such as *Seriola quinqueradiata*^49^, *Anguilla japonica*^50^, and *Paralichthys olivaceus*^51^.

The influences of nutritional BA on fish body structure appear to vary among species, except in terms of lipid content. Zeng, Liao^52^ stated that BA supplementation significantly increased crude protein levels without impacting moisture or ash content. Similarly, Gu, Bai^53^ found that BA had no substantial influence on the ash content, moisture, and crude protein in juvenile turbot. However, Sun, Wang^54^ observed that nutritional BA substantially reduced moisture content while increasing crude protein and ash levels in whole-body, muscle, and liver samples. In the current study, dietary BA substantially impacted crude protein and ash content in whole-body composition, findings similarly reported by Jiang, Wen^55^.

Blood parameters are essential biomarkers that provide insights into fish’s health and physiological status^56^. In this investigation, the hematological and biochemical indicators confirmed that the fish remained healthy throughout the experiment. A high hemoglobin concentration indicates efficient respiratory function and the absence of anemia, a key factor in maintaining fish well-being^57^. Blood measurements are widely used to assess the nutritional adequacy of diets, detect potential toxicity, and evaluate their effects on the circulatory system in aquatic species^58^. Previous investigations have concluded that bile acids (BAs) enhance immune responses in other animals. Hematological and biochemical alterations are commonly utilized to monitor fish healthiness, indicating environmental and dietary changes to which fish are exposed^59,60^. Therefore, BA supplementation significantly influenced the hematological and biochemical markers of *O. niloticus*, demonstrating that the insertion of BAs in the diet had no side effects on fish health. These results align with an earlier investigation by Li, Liu^61^ found that dietary Biogen^®^ and sodium butyrate had no significant impact on hematological parameters.

Dietary BA supplementation also played a crucial role in enhancing immune function, as indicated by improvements in lysozyme activity, immunoglobulin levels (AST), and (ALT) in fish, particularly those receiving suboptimal diets. These suggest that BAs serve as effective growth-enhancing and lipid-reducing feed additives, supporting sustainable aquaculture practices, as Li, Liu^61^ concluded. Furthermore, antioxidant capacity is a key factor in growth performance in fish^62^. Fish-fed BA-enriched diets displayed substantially amplified SOD and CAT activity, along with decreased MDA values in liver tissues, suggesting an enhanced antioxidant defense system. The results suggested that nutritional BAs enhanced the antioxidant capacity of Nile tilapia, similar to observations in mammalian studies where BAs have been shown to enhance antioxidant potential^63^. A stronger antioxidant response may contribute to enhanced growth performance and overall health.

Additionally, fish receiving BA-containing diets demonstrated higher phagocytic activity and index than the control treatment, likely because of the immunostimulatory properties of BAs, which enhance antibacterial defense mechanisms^64^. Immunological improvements detected in the current research align with those described by Ramesh, Vinothkanna^65^, further confirming the BAs role in boosting immune responses. The fundamental humoral immune defense in farmed fish depends on lysozyme activity, which targets bacterial peptidoglycans, particularly in Gram-positive bacteria, leading to bacterial destruction and facilitating phagocytosis by immune cells^58^. Sanjita Devi, Rajiv^66^ similarly found that BA supplementation increased gamma immunoglobulin levels, further strengthening immune function. These findings underscore the capability of BA supplementation to enhance growth efficiency, immune function, and overall physiological health in farmed fish, making it a promising feed additive for sustainable aquaculture.

Intestinal morphometric variables are commonly utilized to assess nutrient absorption efficiency by evaluating villi structure and functionality^67,68^. The length and width of intestinal villi are directly associated with optimal intestinal health, enhanced nutrient absorption, and improved overall fish well-being. In the current investigation, all fish-fed BA-supplemented diets exhibited no signs of intestinal inflammation, suggesting that BA inclusion did not induce any adverse histological effects. Instead, dietary BA supplementation significantly increased villus length and width, absorption area, crypt depth, and the number of goblet cells in the mid-intestine. These results indicate that BA positively influences intestinal health and nutrient absorption, ultimately contributing to improved growth performance in *O. niloticus*. The advantageous properties of BA may be due to its organic acid and salt content, such as sodium butyrate, which has been recognized as a crucial energy source for intestinal epithelial cells^69^ and influenced various cellular functions essential for intestinal integrity^70^.

Additionally, the effectiveness of nutrient absorption is closely linked to villus size^71–73^. Furthermore, dietary BA enhances gastrointestinal tract function by improving absorptive capacity, ensuring more efficient digestion and nutrient uptake. Goblet cells, which have a protective function in the intestinal mucosal layer by secreting mucus and antibacterial substances, aid digestion while reducing pathogenic microorganisms’ prevalence^74^. The present study concluded a substantial increase in goblet cell count in fish-fed BA-enriched diets, reinforcing the beneficial role of BA in promoting gut health and nutrient utilization in *O. niloticus*.

Liver histological examination indicated distinct alterations between the trial group. In the control group (D1), The liver has circular polygonal hepatocytes organized in cord-like formations, bordered on one side by hepatic capillaries or sinusoids. At the core of each cord are slender bile canaliculi located next to the hepatocyte. The liver also exhibited the existence of the pancreatic part (hepatopancreas), which fully encircled the afferent vein. However, in fish given the D2 diet, the liver exhibited subcapsular leukocytic infiltration and marked lytic and cavitation within the hepatic tissues. The liver showed slight cloudy swelling of hepatocytes, and few pyknotic nuclei were detected in the D3 group, where fat deposition altered the typical hepatic architecture, leading to parenchymal fibrosis. The liver exhibits both focal and diffuse vacuolar deterioration of hepatocytes, characterized by severe and widespread vacuoles and focal leucocytic infiltration in D4. The liver exhibits diffuse deterioration of hepatocytes, focal necrosis, leukocytic infiltration, and fat cell deposits in the central vein in diet D5. The liver has significant deterioration characterized by localized necrosis, indicated by pyknotic nuclei and the accumulation of adipocytes, disrupting the liver’s typical architecture, along with parenchymal fibrosis and fat deposition (D6).

Additionally, nuclear migration and hepatocyte vacuolization were prominent in the D1 and D6 treatments. Interestingly, fish-fed BA-enriched diets verified reduced ALT and AST activity, suggesting improved liver function than the control group. These findings contrast with the conclusions of Woolbright, Dorko^75^, who stated that excessive nutritional BA enrichment is hepatotoxic, albeit the processes involved are not well understood. This investigation indicates that moderate BA inclusion in fish diets can improve intestinal healthiness and nutrient absorption without causing detrimental hepatic effects, highlighting its efficacy as a valuable nutritional additive in aquaculture.

In the existing investigation, the mRNA expression levels of ATGL and PPARα in the liver, peritoneal fat, and muscle tissue were meaningfully upregulated in fish fed a diet containing 0.5 g BA/kg compared to other groups. Additionally, the expression of *HSL* in intraperitoneal fat was elevated in the same group. Both ATGL and HSL are critical lipid-catabolizing enzymes responsible for lipid hydrolysis^76^, with ATGL functioning as the rate-limiting enzyme in triglyceride (TG) breakdown^77^. The hydrolysis of TG is initiated by ATGL, which converts it into diglycerides and free fatty acids^78^. Subsequently, HSL further breaks down diglycerides into monoglycerides and additional free fatty acids^79^. The upregulation of HSL and ATGL induced by dietary BA suggests an enhancement in lipid breakdown and mobilization within these organs. The results align with the observations of Haemmerle, Moustafa^80^, who documented elevated PPARα expression in fish consuming a diet enriched with 1.5 g BA/kg. PPARα is a nucleotide receptor that regulates genes related to fatty acid metabolism, including transfer and oxidization, notably in the liver, where it facilitates hepatic fatty acid oxidation^81^.

The enzyme CPT^−1^ enables the transference of long-chain fatty acids across mitochondrial membranes for β-oxidation^82^. Associated with the D2 and D3 groups, fish fed the D4 diet exhibited higher CPT^−1^ expression in intraperitoneal fat tissue than in the liver or muscle, suggesting that mitochondrial β-oxidation was more active in adipose tissue than other organs. Conversely, CPT^−1^ expression values were highest in the muscle of fish given the D2 diet, which contained higher lipid levels, probably due to amplified lipid storage in muscle relative to other dietary treatments. Fatty acid synthase is vital in de novo lipogenesis, catalyzing the alteration of acetyl-CoA and malonyl-CoA into palmitate^83^. In the present examination, FAS expression was lowest in the liver, fat, and muscle of D2-fed fish among the other groups. This finding aligns with research demonstrating that nutritional lipids regulate FAS’s activity and mRNA expression^84^. Interestingly, fas expression levels in intraperitoneal fat and muscle were comparable in fish receiving 0.5 g BA/kg, indicating that BA supplementation did not influence de novo fatty acid synthesis. However, regulating FAS is a multifaceted process affected by several variables, particularly glucocorticoids, insulin, and leptin^85^. Thus, the differences between the present findings and those reported by^86^ may be attributed to additional regulatory mechanisms beyond those considered in this study.

In addition to analyzing lipid metabolism-related genes, this study also examined the expression of (Igf^−II^), a gene associated with protein metabolism in *O. niloticus* fed experimental diets. Igf^−II^ fundamentally regulates growth and development^87^. Under the current experimental conditions, Igf^−II^ expression was meaningfully upregulated in the muscle of fish-fed BA-enriched diets compared to the control group, suggesting a potential role of BA in promoting muscle growth and protein metabolism. Interestingly, the published research shows numerous variables in findings, including the types and concentrations of BAs utilized in diet formulation, the diet components used as basal constituents (diets based on proteins, lipids, or carbohydrates), and fish species. It is important to highlight that these results may show how dietary BA may help mitigate the detrimental effects of a diet high in fat on liver function at a certain concentration.

## Conclusion

This investigation revealed that nutritional bile acid (BA) 0.2 g/kg supplementation effectively promotes growth in *Oreochromis niloticus*, potentially improving lipase secretion, which facilitates lipid digestion and absorption. In addition to improving growth performance, BA supplementation positively influenced proximate body composition, hematological parameters, non-specific immune responses, and antioxidant activity. Furthermore, gene expression analysis revealed significant upregulation of key metabolic genes in fish-fed BA-enriched diets. Histological examination also indicated improvements in intestinal morphometry and liver structure, further supporting the beneficial effects of BA in enhancing nutrient utilization and overall physiological health in Nile tilapia. These results recommend that BA addition could be an important strategy for enhancing fish health and well-being in aquaculture.

## Data Availability

Data is available from the corresponding author upon reasonable request.
